# Outbreak of cutaneous leishmaniasis before and during the COVID-19 pandemic in Jahrom, an endemic region in the southwest of Iran

**DOI:** 10.1080/22221751.2022.2117099

**Published:** 2022-09-28

**Authors:** Samaneh Mazaherifar, Kavous Solhjoo, Amir Abdoli

**Affiliations:** a Zoonoses Research Center, Jahrom University of Medical Sciences, Jahrom, Iran; b Department of Parasitology and Mycology, Jahrom University of Medical Sciences, Jahrom, Iran

**Keywords:** SARS-CoV-2, *Leishmania major*, zoonotic cutaneous leishmaniasis, Iran, COVID-19

## Abstract

The emergence of the Coronavirus Disease 2019 (COVID-19) pandemic has a considerable effect on the burden of other diseases. Cutaneous leishmaniasis (CL) is an endemic parasitic disease in Iran. Here, we report an outbreak of zoonotic cutaneous leishmaniasis (ZCL) during the COVID-19 pandemic in Jahrom county, which is an endemic region in the southwest of Iran. Before the pandemic, the annual occurrence of CL was less than 240 cases per year, while the number of cases increased to 307 and 771 cases in the first and second years after the pandemic, respectively. Molecular detection of some isolates identified *Leishmania major*. The rodent control program was completely interrupted during the first year of the COVID-19 outbreak in Jahrom (February to December 2020), then the program restarted again as routine from the summer of 2021 till now. Interrupted rodent control program along with inadequate screening programs of CL patients were probably one of the causes of this outbreak in Jahrom.

Leishmaniasis is an endemic vector-borne disease in the tropical and subtropical regions of the world [1], including Iran [2]. Cutaneous leishmaniasis (CL) and visceral leishmaniasis (VL) are the two most common forms of the disease. CL is endemic in different parts of Iran with an incidence rate of about 1.18 and 5.7 Disability Adjusted Life Years (DALYs) per 100,000 population [3]. Zoonotic cutaneous leishmaniasis (ZCL) (caused by *Leishmania major*) and anthroponotic cutaneous leishmaniasis (ACL) (caused by *L. tropica*) are the two main causative agents of CL in Iran [3].

The emergence of Coronavirus Disease 2019 (COVID-19) has a considerable effect on the burden of communicable [4] and non-communicable diseases [5] throughout the world and in Iran [6]. Iran is among the countries that were severely affected by the COVID-19 pandemic [6]. The first confirmed cases of COVID-19 were officially reported on 19 February 2020 in Iran [7].

We report a new surging of ZCL in an endemic region of Iran (Jahrom, Fars Province, southwestern Iran; Coordinates: 28°30′00″N 53°33′38″E, Figure 1A). We retrospectively obtained data from individuals with CL who were referred to the reference laboratory of leishmaniasis (Ghafoori Laboratory) in the city of Jahrom from April 2016 until 20 March 2022. Data of the confirmed cases of CL were gathered from the patient medical records, who were diagnosed by light microscope following direct smear of the lesions and Giemsa staining. Moreover, during the pandemic period, we obtained samples from 117 patients for molecular identification of the *Leishmania* species by nested-PCR targeting the kinetoplast DNA (*kDNA*) gene [8]. Furthermore, five isolates from Jahrom county and five isolates from other parts of Iran were selected for sequencing by the internal transcribed spacer-1 (*ITS*-1) gene [8].

**Figure 1.**
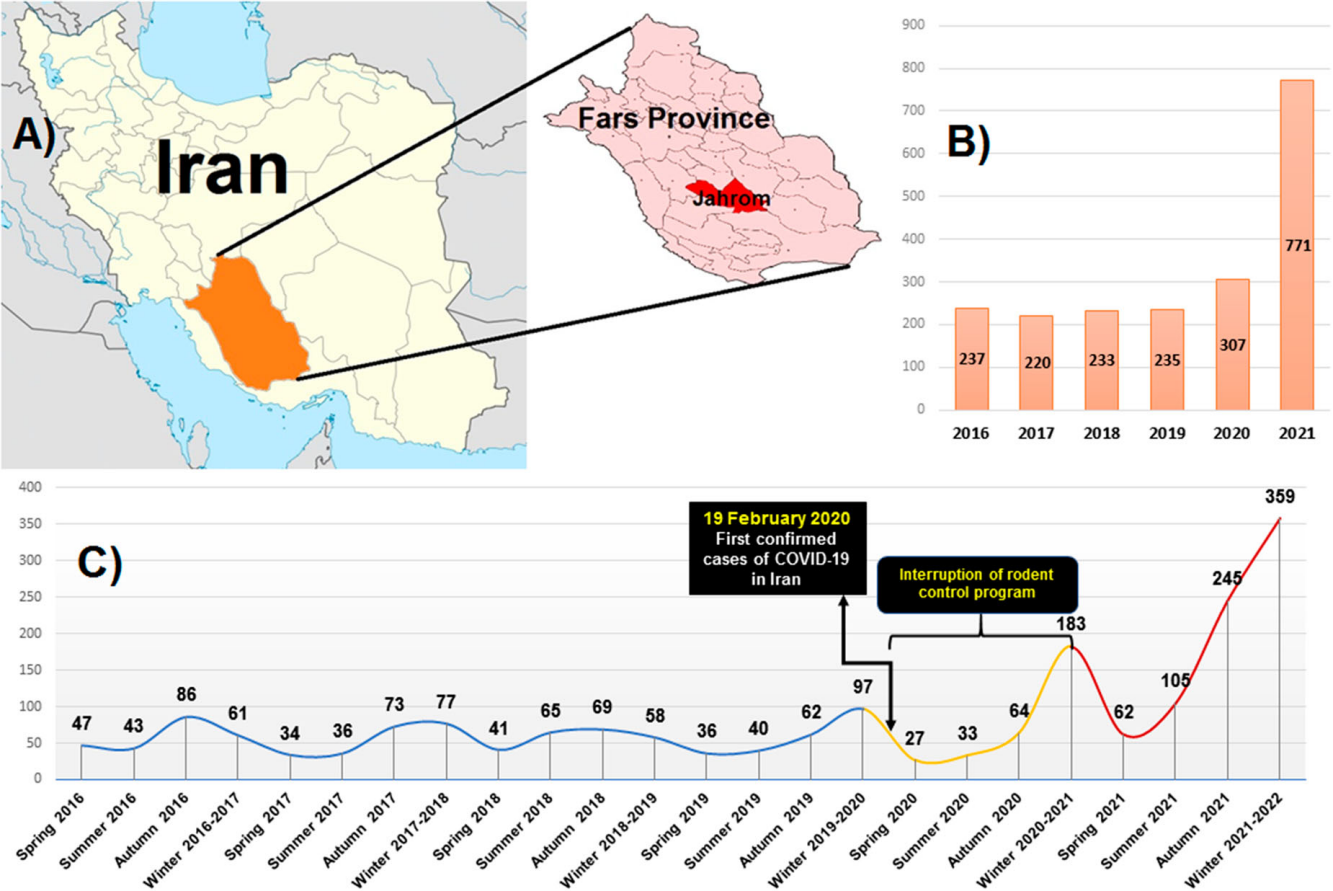
(A) The map of Iran and the outbreak region; (B) yearly trends of cutaneous leishmaniasis occurrence in Jahrom county; (C) trends of cutaneous leishmaniasis-based season from Spring 2016 to Winter 2021–2022. Note that the blue line is the trends before the COVID-19 pandemic, the yellow and red lines are the first and second years after the pandemic. The black boxes demonstrate the first confirmed case of COVID-19 in Iran and the time of rodent control program interruption.

The yearly trends of CL occurrences have shown a steady trend from spring 2016 till the end of 2019 (237, 220, 233, and 235 cases from 2016, 2017, 2018, and 2019, respectively), while a slightly increased was observed in 2020 (307 cases), and a sharp peak with more than twofold than 2019 has emerged at the end of 2021 (771 cases) (Figure 1B). As shown in Figure 1(C), the lowest number of cases was recorded in spring followed by summer, while the peak prevalence was in autumn followed by winter. Notably, the lowest prevalence seasons (from 2016 to 2022) were in the spring and summer of 2020, whereas the first peak occurred in autumn 2020 and the second sharp peak emerged in autumn 2021 and winter 2021–2022. Demographic characterization of the patients (1078 cases) after the pandemic revealed that 56.7% and 43.3% of the patients were males and females, respectively. Age ranges of the patients were as followed: <10: 20.31%, 11–20: 9.09%, 21–30: 10.29%, 31–34: 20.12% and >41: 40.16%. Most cases had a single lesion (55.47%), while two, three, and multiple lesions were observed in 16.14%, 15.12%, and 13.26% of the cases, respectively. Furthermore, most lesions were on hands (52.69%), while 16.88%, 9.18%, and 21.24% of the lesions were observed on the legs, face, and multiple locations, respectively.

The results of PCR revealed that all isolated were diagnosed as *L. major*, and sequencing confirmed this result (GenBank accession numbers: OL627363-72) [8]. Previous studies before the pandemic revealed that both ZCL and ACL were frequent in Jahrom. In this regard, Davami et al. obtained samples from 40 cases of CL during 2008 to determine the type of *Leishmania* species by nested-PCR targeting the *kDNA* gene in Jahrom. The results identified *L. major* and *L. tropica* in 87.5% and 12.5% of clinical isolates [9]. In another study in the same region, Rahmanian et al. [10] obtained samples from 165 cases of CL during 2017 and determined the species of the parasite by nested-PCR targeting the *kDNA* gene. The results identified *L. major* and *L. tropica* in 98.8% and 1.2% of the samples, respectively [10]. Whereas all isolates of this study identified as *L. major*, it seems that the pattern of CL has been changed to *L. major* in Jahrom. While ZCL is zoonotic and rodents are the main reservoir of this type, rodent control could be among the first preventive interventions for the control of ZCL.

Several actions were performed to prevent the spread of COVID-19 immediately after the first surge of the disease in Iran (23 March 2020), including mandatory mask use policy, closure of schools, offices, judiciary system, and religious places (e.g. mosques and holy shrines) [6, 11]. During the COVID-19 outbreak, the health system of Iran was severely affected and most budget was assigned to the COVID-19 management [6]. In Jahrom County, the rodent control program is routinely performed by rodenticide four or five times a year during spring and summer. During the first year of the COVID-19 outbreak in Iran (February to December 2020), the rodent control program was completely ceased, then the program restarted again from the summer of 2021 till now. Although the rodent control program was restarted since spring 2021, a sharp peak of CL occurrence was seen in autumn and winter 2021 (Figure 1C). Jahrom is an agricultural pole in Iran, and its most important agricultural products are dates and citrus. As such, most people in Jahrom inhabited houses that usually have a courtyard with citrus and date trees. These conditions provide a suitable circumstance for rodent and sandfly propagation. Although the restriction of outdoor activities may help to decrease the rate of CL, the environment of Jahrom County provides a suitable condition for reservoirs and vectors of *L. major* (rodents and sandflies) that facilitate the transmission of the parasite to humans. Hence, the interrupted rodent control program alongside with inadequate screening programs were probably one of the causes of this outbreak in Jahrom.

Previous reports in Iran demonstrated that the implementation of rodent control is an effective strategy for the control of ZCL [12–14]. As such, the implementation of health education programs for inhabitants of endemic regions could help to increase their knowledge, attitude, and performance regarding prevention practices of leishmaniasis [15, 16]. Hence, these actions could be recommended for the control of CL in Jahrom County as well as other endemic regions.

## Limitations of the study

We could not obtain a history of COVID-19 infection from the CL patients during the pandemic. Indeed, we could not attain data from other endemic regions of Iran to compare the occurrence of CL before and after the pandemic. Therefore, these topics should be considered to evaluate in future investigations.

## Ethics approval

All participants were informed about the study, and sampling was conducted with the informed consent of the patients or their parents. The study protocol was approved by the Research and Ethics committee of the Jahrom University of Medical Sciences, Jahrom, Iran (ethics code: IR.JUMS.REC.1399.114), and National Institute for Medical Research Development (NIMAD) (the ethics code: IR.NIMAD.REC.1398.249).
